# Genetic Influences on Hand Osteoarthritis in Finnish Women – A Replication Study of Candidate Genes

**DOI:** 10.1371/journal.pone.0097417

**Published:** 2014-05-13

**Authors:** Satu Hämäläinen, Svetlana Solovieva, Tapio Vehmas, Katariina Luoma, Päivi Leino-Arjas, Ari Hirvonen

**Affiliations:** 1 Finnish Institute of Occupational Health, Centre of Expertise for Health and Work Ability, Helsinki, Finland; 2 University of Helsinki, Helsinki University Central Hospital, Radiology department, Helsinki, Finland; National Cancer Institute, National Institutes of Health, United States of America

## Abstract

**Objectives:**

Our aims were to replicate some previously reported associations of single nucleotide polymorphisms (SNPs) in five genes (A2BP1, COG5, GDF5, HFE, ESR1) with hand osteoarthritis (OA), and to examine whether genes (BCAP29, DIO2, DUS4L, DVWA, HLA, PTGS2, PARD3B, TGFB1 and TRIB1) associated with OA at other joint sites were associated with hand OA among Finnish women.

**Design:**

We examined the bilateral hand radiographs of 542 occupationally active Finnish female dentists and teachers aged 45 to 63 and classified them according to the presence of OA by using reference images. Data regarding finger joint pain and other risk factors were collected using a questionnaire. We defined two hand OA phenotypes: radiographic OA in at least three joints (ROA) and symptomatic DIP OA. The genotypes were determined by PCR-based methods. In statistical analysis, we used SNPStats software, the chi-square test and logistic regression.

**Results:**

Of the SNPs, rs716508 in *A2BP1* was associated with ROA (OR = 0.7, 95% CI 0.5–0.9) and rs1800470 in *TGFB1* with symptomatic DIP OA (1.8, 1.2–2.9). We found an interaction between ESR1 (rs9340799) and occupation: teachers with the minor allele were at an increased risk of symptomatic DIP OA (2.8, 1.3–6.5). We saw no association among the dentists. We also found that the carriage of the *COG5* rs3757713 C allele increased the risk of ROA only among women with the *BCAP29* rs10953541 CC genotype (2.6; 1.1–6.1). There was also a suggestive interaction between the *HFE* rs179945 and the *ESR1* rs9340799, and the carriage of the minor allele of either of these SNPs was associated with an increased risk of symptomatic DIP OA (2.1, 1.3–2.5).

**Conclusions:**

Our results support the earlier findings of *A2BP1 and TBGF1* being OA susceptibility genes and provide evidence of a possible gene-gene interaction in the genetic influence on hand OA predisposition.

## Introduction

Osteoarthritis (OA) shows clinical heterogeneity in localization and progression [1]. Patients may have only one affected joint (monoarthritis) at the time of diagnosis, several affected joints within a single region (*e.g.* in hand OA), or several involved joints at various sites, *e.g.* the hip, knee, and hand (polyarticular OA, generalized OA). Twin and family studies have demonstrated a significant contribution of genetic factors that account for up to half of the risk of developing OA [2], [3].

The hand is among the most prevalent sites affected by OA, especially among women over the age of 50 [4]. The simultaneous involvement of multiple hand joints makes hand OA a heterogeneous disorder that is complex to study [5]. Hand OA, largely mirroring the generalized OA variant, is thought to be more heritable than hip or knee OA [3], [6]. It is generally accepted that as a complex disorder, the development of hand OA is modulated by many genes with small effects and gene-environment interaction. The genetic influence may involve either a structural defect (*e.g*., in collagen), alterations in the structural extracellular matrix (ECM) proteins of cartilage and bone, an enhanced inflammatory component in the disease process, or a genetic influence on a known risk factor for OA, such as obesity [7].

Genome-wide scans with different hand OA phenotypes suggested that chromosomes 1, 2, 3, 4, 7, 8, 9, 11, 13, 15, 16, 19, 20 may harbor susceptibility genes [8]–[13]. Numerous candidate gene studies have been carried out to assess the association of a particular variant with hand OA. However, according to a systematic review, specific associations between a gene and hand OA have rarely been analyzed by more than one study, and only for two genes (*AGC1* and *HFE)* have significant associations been replicated by at least two independent studies [14].

A genome-wide association study (GWAS) is a promising tool for discovering the genetic basis of common diseases [15]. GWAS were successful in the identification of 11 loci associated with different OA phenotypes, in particular knee and hip OA [16]. Nevertheless, under the strict criteria of replication and a known functional role, the growth and differentiation factor 5 (GDF5) is the only truly OA-associated gene at present [17]. To our knowledge only two GWAS of hand OA have been published so far [18], [19].

The first GWAS study detected and replicated an association with an SNP (rs716508) in the first intron of the ataxin 2-binding protein 1 gene (A2BP1) [18]. In the second GWAS, a novel common variant (rs3815148) in intron 12 of the component of the oligomeric Golgi complex 5 gene (COG5) was associated with knee and/or hand OA in both discovery and replicated samples. This SNP is in almost complete linkage disequilibrium with rs3757713 (68 kb upstream of GPR22), which could be the link to OA association. In the same GWAS, three loci in the *GDF5* gene (rs4911494, rs6088813, rs6087705) were associated with hand OA in the discovery sample, but were not replicated [19].

In order to devise future prevention and treatment, strategies for OA replication studies that verify positive findings from GWAS are needed. Such studies will also enable us to determine whether the effect is specific to a certain OA phenotype, and/or to a particular population. Furthermore, GWAS have made it evident that most of the genetic risk of complex diseases will be attributed to numerous genes with small to moderate effect sizes [16]. In order to understand the manner in which the individual genes that are implicated in OA exert their effect, gene-gene combination effect and interaction need to be explored.

Our aims were to replicate some previously reported associations with hand OA for single nucleotide polymorphisms (SNPs) in five genes (A2BP1, COG5, GDF5, HFE, ESR1) and to examine whether variants in nine genes (BCAP29, DIO2, DUS4L, DVWA, HLA, PTGS2, PARD3B, TGFB1 and TRIB1) are associated with hand OA among Finnish middle-aged women. All selected SNPs were located on genes with prior evidence from GWAS or candidate gene association studies of an association with different OA phenotypes.

## Participants and Methods

### Study Population

The study participants were identified from the registers of the Finnish Dental Association and the Finnish Teachers Trade Union, and randomly selected from both occupational groups by using the place of residence (Helsinki or its neighboring cities) as an inclusion criterion. The samples were restricted to women aged 45 to 63. Of those who received the questionnaires in 2002, 295 (68%) dentists and 248 (57%) teachers participated in a clinical examination.

### Ethics Statement

Participation in the study was voluntary and based on written informed consent. The study proposal was approved by the Hospital District of Helsinki and Uusimaa Ethics Committee for Research in Occupational Health and Safety.

### Hand Radiography and Image Analysis

Both hands of the study participants were radiographed by exposing Kodak X-ray films with Siemens X-ray equipment (48 kV, 10 mA, focus film distance = 115 cm; Siemens, Munich, Germany). The analogue radiographs were evaluated by an experienced radiologist who was blinded to the occupation, age, and all health data of the participants. Each distal interphalangeal (DIP), proximal interphalangeal (PIP), thumb interphalangeal, and metacarpophalangeal (MCP) joint of both hands was graded separately, and classified for the presence of OA using a modified Kellgren and Lawrence system [20]. The classification criteria were: grade 0 = no OA, grade 1 = doubtful OA, grade 2 = mild OA, grade 3 = moderate OA, and grade 4 = severe OA. Reference images were used; their description is given elsewhere [21]. A second experienced radiologist interpreted 46 randomly chosen radiographs. A second reading of these 46 radiographs was independently performed by the primary radiologist (TV). The reliability of the OA classification was estimated by measuring intra-observer and inter-observer agreements using the weighted Cohen’s kappa coefficient with quadratic weights [22]. The inter-observer agreement for OA ranged from 0.67 to 0.85 (good) for DIP joints, from 0.39 to 0.61 (moderate) for PIP joints, and from 0.18 to 0.69 (poor to good) for MCP joints. The intra-observer agreement for OA ranged from good to very good, 0.73 to 0.88 for DIP joints, from 0.67 to 0.92 for PIP joints, and from 0.59 to 1.0 for MCP joints. [21].

### Questionnaires and Interviews

All study participants received a self-administered questionnaire that included questions on anthropometric measures. Information on symptoms (pain, tenderness) in each joint studied was collected with the prompt: ‘Please point out on the picture below in which finger joint you have felt pain or tenderness during the past 30 days.’ The participants were also asked to mark the intensity of the pain: 0 = no pain, 1 = mild pain, 2 = moderate pain, 3 = severe pain. Weight was measured without shoes to the accuracy of 0.1 kg. Body mass index (BMI) (weight (kg) per height squared (m^2^)) was calculated on the basis of self-reported height and measured weight.

### Hand OA Phenotypes

Two hand OA phenotypes were used. If the participant had radiographic findings (grade ≥2) in at least three finger joints she was classified as having radiographic OA (ROA). Otherwise, the participant was classified as not having ROA. If the participant had both radiographic findings (grade ≥2) and symptoms (grade ≥1) in at least two DIP joints, she was classified as having symptomatic DIP OA. Otherwise, the subject was classified as not having symptomatic DIP OA.

### SNP Selection

We aimed primarily to replicate the associations for the candidate genes that have been identified by two recent GWAS of hand OA [18], [19] and genes with a known association with hand OA pathology. We chose two SNPs (rs716508 and rs3815148) that reached a genome-wide level of significance for association with hand OA [18], [19], and variants in the GDF5 (rs143383) [23], HFE (rs1799945) [24], and ESR1 (rs2234693, rs9340799) [25], [26] genes. The latter genes were chosen on the basis of the rapidly increasing prevalence of hand OA in women over the age of 45. In addition, we searched PubMed for studies reporting an association between any OA phenotype and the candidate genes located on the chromosomes identified by genome-wide linkage studies as harboring susceptibility genes for hand OA [8]–[13] (Leppävuori et al. 1999, Demissie et al. 2002, Stefánsson et al. 2003, Hunter et al. 2004, Greig et al. 2006, Livshits et al. 2007). Studies published by 10.11.2010 were reviewed in order to select relevant SNPs. Whenever at least one significant functional SNP was reported for a given candidate gene in hand OA, the SNP was selected for analyses in our samples. Finally, we selected variants from nine candidate genes (BCAP29, DIO2, DUS4L, DVWA, HLA, PTGS2, PARD3B, TGFB1 and TRIB1) from seven publications [27]–[33] for exploratory analysis in our study. Several predicted functional effects were found in the F-SNP database for the studied polymorphisms [34]. The description of SNPs selected for replication is given in Table S1.

### Genotyping Analysis

Blood samples were taken from each study participant at the clinical examination and stored at +4°C until we extracted DNA using a DNA extraction kit (PUREGENE DNA Purification Kit; Gentra Systems, Plymouth, MN, USA).

The ESR1 *PvuII*, and *Xbal* polymorphisms (rs2234693, and rs9340799 respectively) were genotyped using the RFLP method as essentially described in [35]. The *TGFB1* Leu10Pro (29T>C, rs1800470 formerly known as rs1982073) polymorphism was genotyped by the TaqMan PCR method as described in [36] with somewhat altered PCR conditions (2 min +50°C, 10 min +95°C and 40 cycles 15 s +95°C, 1 min +62°C).

We analyzed the rest of the studied polymorphisms in A2BP1 (rs716508), BCAP29 (rs10953541), COG5 (rs3757713 and rs3815148), PTGS2 (rs4140564), DIO2 (rs225014), DUS4L (rs4730250), DVWA (rs7639618), GDF5 (rs143383), HFE (rs1799945), HLA (rs10947262), PARD3B (rs1207421) and TRIB1 (rs4512391) simultaneously using OpenArray equipment and the TaqMan SNP Genotyping Assays (Applied Biosystems, C__1224093_10, C__2618842_20, C_27475119_10, C_25994114_10, C_31274663_20, C_15819951_10, C_32373604_10, C__1176713_10, C__1270479_1, C__1085600_10, C_32201424_10, C__8807483_10 and C___310264_20, respectively).

Genotyping completion rate was from 99.8 to 100%.

### Statistical Analysis

The potential deviation of the allele frequencies from the Hardy-Weinberg equilibrium (HWE) was tested from total population using the chi-square test. The degree of pairwise linkage disequilibrium (LD) for two SNPs in the *COG5* gene and two SNPs in the *ESR1* gene were calculated using SNPStats software [37]. Each SNP was analyzed in turn. Haplotypes were constructed and analyzed by the SNPStats program. Logistic regression analysis was used to test the associations between selected SNPs and two hand OA phenotypes. For each SNP, a log additive model of inheritance was fitted. Gene-occupation interaction was tested for all SNPs, to evaluate whether the association between the SNPs and OA was modified by occupation. In addition, gene-gene interactions and gene-gene combination effects were evaluated by a logistic regression model with a dummy variable (0, 1) for the SNPs to compare the magnitude of their odds ratios (ORs). From each gene, we selected a representative SNP that showed the lowest p-value in the replication analysis. We used the dominant model, with the homozygous genotype of the major allele as the reference. Both crude and adjusted ORs and their 95% confidence intervals were calculated. We adjusted the ORs for the potential confounding effects of age (continuous), occupation (dentists vs. teachers) and BMI (continuous). Since the crude and adjusted ORs did not differ significantly, only the adjusted ORs are shown.

All analyses were hypothesis driven. We have used p<0,05and p<0.008 as significance level in replication and exploration analyses, respectively. In exploratory analyses p-values were adjusted for multiple testing using Sidák’s method [38]. We used SNPStats software [37] for the analyses.

## Results

The prevalence of ROA and symptomatic DIP OA were 29.5%, and 9.0%, respectively. The ROA prevalence was statistically significantly higher among the teachers than the dentists (Table 1). The genotype distributions of the selected SNPs did not deviate from the HWE. There was no difference between the minor allele frequencies for any SNPs of the two occupations.

**Table 1:** Selected characteristics of study population.

	Radiographic OA*		Symptomatic DIP OA**	
	No	Yes	No	Yes
**All (n = 542)**	382 (70.5%)	160 (29.5%)	493 (91.0%)	49 (9.0%)
Age (mean ± SD)	53.0±5.2	56.3±4.7	53.6±5.2	57.7±4.2
BMI (mean ± SD)	24.3±3.5	25.0±3.8	24.4±3.6	25.1±3.3
Pinch grip strength(mean ± SD)	55.9±7.4	52.3±9.2	55.2±8.1	51.5±7.0
**Dentists (n = 294)**	222 (75.5%)	72 (24.5%)	274 (93.2%)	20 (6.8%)
Age (mean ± SD)	52.6±5.6	57.1±5.6	53.3±5.8	59.4±4.8
BMI (mean ± SD)	23.8±3.2	24.5±3.3	23.9±3.3	23.9±3.0
Pinch grip strength(mean ± SD)	56.3±7.5	52.7±10.1	55.8±8.2	50.4±8.4
**Teachers (n = 248)**	160 (64.5%)	88 (35.5%)	219 (88.3%)	29 (11.7%)
Age (mean ± SD)	53.5±4.5	55.7±3.8	53.9±4.5	56.5±3.3
BMI (mean ± SD)	25.0±3.8	25.4±4.1	25.0±4.0	25.9±3.3
Pinch grip strength(mean ± SD)	55.3±7.1	52.0±8.4	54.4±8.0	52.2±5.8

*p = 0.006 for difference between prevalence among dentists and among teachers.

**p = 0.05 for difference between the prevalence among dentists and among teachers.

### Replication Analyses of Hand OA-associated SNPs

Of the two SNPs identified in GWAS, only the rs716508 located in the *A2BP1* gene was associated with ROA (OR = 0.68, 95% CI 0.50–0.93) (Table 2). However it was not associated with symptomatic DIP OA (Table 3). The risk of symptomatic DIP OA was marginally, though not statistically significantly, elevated with the occurrence of the minor alleles of two SNPs (rs3757713, rs3815148) in the *COG5* gene.

**Table 2:** Association of studied polymorphisms with ROA in the total material and by occupation.

Gene	Chr	SNP	MAF (%)		Group	MAF (%) Our study			HWE	ROA		
			NCBI	HapMap		Total	Case	Control	(p-value)	OR	95% CI	p-value
**Replication analyses**												
A2BP1	16	rs716508	39	28	All	35	31	36	0.09	**0.68**	**0.50–0.93**	**0.01**
					Dentists	35	33	36		0.69	0.39–1.23	0.19
					Teachers	34	28	37		0.58	0.34–1.01	0.05
COG5	7	rs3757713	14	21	All	19	20	18	0.07	1.16	0.83–1.63	0.38
					Dentists	19	23	18		1.60	0.90–2.86	0.09
					Teachers	18	18	18		0.97	0.54–1.75	0.92
COG5	7	rs3815148	19	22	All	19	20	18	0.12	1.14	0.81–1.59	0.45
					Dentists	19	24	18		1.51	0.84–2.71	0.11
					Teachers	18	17	18		0.90	0.50–1.62	0.74
GDF5	20	rs143383	29	32	All	43	41	43	0.55	0.93	0.70–1.22	0.58
					Dentists	43	40	44		0.80	0.44–1.43	0.49
					Teachers	42	42	42		1.21	0.68–2.14	0.51
HFE	6	rs1799945	8	18	All	13	13	12	0.53	1.15	0.76–1.74	0.52
					Dentists	12	10	12		0.86	0.43–1.74	0.68
					Teachers	14	15	13		1.55	0.84–2.88	0.19
ESR1	6	rs2234693	44	41	All	40	39	41	0.60	0.94	0.71–1.24	0.67
					Dentists	41	40	41		0.80	0.45–1.43	0.45
					Teachers	40	39	40		1.08	0.62–1.89	0.82
ESR1	6	rs9340799	26	30	All	25	26	25	0.20	1.05	0.76–1.45	0.78
					Dentists	27	26	27		0.83	0.47–1.45	0.51
					Teachers	24	26	23		1.62	0.94–2.79	0.08
**Exploratory analyses**												
PTGS2	1	rs4140564	4	8	All	7	6	7	0.35	0.90	0.50–1.61	0.72
					Dentists	6	3	7		0.40	0.13–1.21	0.08
					Teachers	8	9	7		1.44	0.69–3.02	0.33
PARD3B	2	rs1207421	13	9	All	10	11	9	1.00	1.04	0.66–1.64	0.87
					Dentists	10	12	9		1.17	0.59–2.33	0.67
					Teachers	10	10	9		0.90	0.45–1.82	0.80
DVWA	3	rs7639618	26	18	All	16	16	16	0.95	1.11	0.76–1.63	0.58
					Dentists	18	19	17		1.52	0.84–2.76	0.16
					Teachers	14	14	14		0.82	0.44–1.52	0.57
HLA	6	rs10947262	17	6	All	14	15	14	0.68	1.06	0.72–1.56	0.78
					Dentists	15	16	14		1.10	0.59–2.05	0.82
					Teachers	14	14	14		1.01	0.54–1.89	0.99
BCAP29	7	rs10953541	14	27	All	26	27	25	0.18	1.09	0.81–1.48	0.58
					Dentists	27	29	26		1.43	0.81–2.51	0.18
					Teachers	24	25	24		1.06	0.61–1.83	0.78
DUS4L	7	rs4730250	12	17	All	15	17	15	0.23	1.15	0.80–1.66	0.46
					Dentists	16	20	14		1.63	0.89–2.97	0.10
					Teachers	14	14	15		0.80	0.43–1.49	0.49
TRIB1	8	rs4512391	38	41	All	26	25	27	0.95	0.87	0.64–1.20	0.40
					Dentists	27	28	27		1.17	0.66–2.04	0.58
					Teachers	25	22	26		0.75	0.44–1.31	0.32
DIO2	14	rs225014	42	39	All	28	27	29	0.10	0.85	0.62–1.17	0.32
					Dentists	28	30	27		1.05	0.60–1.85	0.88
					Teachers	28	24	31		0.72	0.42–1.23	0.24
TGFB1	19	rs1800470/rs1982073	44	NA	All	29	31	29	0.58	1.15	0.85–1.57	0.36
					Dentists	31	29	31		1.10	0.63–1.93	0.71
					Teachers	28	32	25		1.29	0.75–2.23	0.36

Logistic regression analysis with log-additive model in group “All”, and dominant model in groups “Dentists” and “Teachers”.

ORs and 95% CIs are adjusted for age, occupation and body mass index (BMI) in group “All” and for age and BMI in groups “Dentists” and “Teachers”.

**Table 3:** Association of studied polymorphisms with symptomatic DIP OA in the total material and by occupation.

Gene	Chr	SNP	MAF (%)		Group	MAF (%)This study			HWE	Symptomatic DIP OA		
			NCBI	HapMap		Total	Case	Control	(p-value)	OR	95% CI	p-value
**Replication analyses**												
A2BP1	16	rs716508	39	28	All	35	35	35	0.09	0.94	0.58–1.52	0.80
					Dentists	35	38	35		0.69	0.26–1.81	0.38
					Teachers	34	33	34		1.06	0.47–2.38	0.94
COG5	7	rs3757713	14	21	All	19	24	18	0.07	1.46	0.90–2.37	0.13
					Dentists	19	23	19		1.35	0.51–3.54	0.62
					Teachers	18	24	17		1.81	0.80–4.12	0.16
COG5	7	rs3815148	19	22	All	19	25	18	0.12	1.35	0.83–2.19	0.24
					Dentists	19	23	19		1.37	0.52–3.59	0.60
					Teachers	18	26	17		1.41	0.61–3.23	0.42
GDF5	20	rs143383	29	32	All	43	39	43	0.55	0.83	0.54–1.29	0.41
					Dentists	43	38	43		0.76	0.29–2.00	0.50
					Teachers	42	40	43		0.74	0.32–1.69	0.50
HFE	6	rs1799945	8	18	All	13	15	12	0.53	1.49	0.80–2.76	0.22
					Dentists	12	18	11		2.29	0.83–6.29	0.11
					Teachers	14	14	14		1.41	0.57–3.49	0.48
ESR1	6	rs2234693	44	41	All	40	45	40	0.60	1.27	0.83–1.94	0.28
					Dentists	41	45	40		1.33	0.48–3.70	0.56
					Teachers	40	45	39		1.47	0.62–3.46	0.38
ESR1	6	rs9340799	26	30	All	25	31	25	0.20	1.39	0.86–2.24	0.19
					Dentists	27	23	27		0.50	0.19–1.33	0.15
					Teachers	24	36	22		**2.84**	**1.25–6.48**	**0.01**
**Exploratory analyses**												
PTGS2	1	rs4140564	4	8	All	7	4	7	0.35	0.53	0.18–1.56	0.22
					Dentists	6	0	6		–	–	–
					Teachers	8	7	8		0.83	0.26–2.64	0.73
PARD3B	2	rs1207421	13	9	All	10	10	10	1.00	0.91	0.45–1.87	0.80
					Dentists	10	10	10		0.90	0.28–2.93	0.84
					Teachers	10	10	10		0.79	0.27–2.25	0.68
DVWA	3	rs7639618	26	18	All	16	14	16	0.95	0.93	0.50–1.71	0.81
					Dentists	18	13	18		0.81	0.27–2.36	0.70
					Teachers	14	16	14		1.02	0.42–2.52	0.97
HLA	6	rs10947262	17	6	All	14	13	14	0.68	0.90	0.48–1.68	0.74
					Dentists	15	20	14		1.78	0.67–4.71	0.25
					Teachers	14	9	14		0.59	0.21–1.64	0.32
BCAP29	7	rs10953541	14	27	All	26	32	25	0.18	1.36	0.86–2.15	0.19
					Dentists	27	28	27		1.07	0.42–2.77	0.99
					Teachers	24	35	23		1.64	0.73–3.65	0.21
DUS4L	7	rs4730250	12	17	All	15	20	15	0.23	1.44	0.84–2.45	0.19
					Dentists	16	20	16		1.76	0.66–4.65	0.31
					Teachers	14	21	14		1.49	0.63–3.50	0.34
TRIB1	8	rs4512391	38	41	All	26	20	27	0.95	0.67	0.39–1.15	0.14
					Dentists	27	25	28		0.99	0.39–2.53	0.96
					Teachers	25	17	26		0.53	0.22–1.23	0.13
DIO2	14	rs225014	42	39	All	28	31	28	0.10	1.13	0.69–1.85	0.63
					Dentists	28	35	27		1.76	0.66–4.68	0.28
					Teachers	28	28	29		0.92	0.41–2.04	0.79
TGFB1	19	rs1800470/rs1982073	44	NA	All	29	40	28	0.58	**1.84**	**1.16**–**2.91**	**0.01**
					Dentists	31	40	30		1.57	0.60–4.09	0.36
					Teachers	28	40	26		**2.72**	**1.16**–**6.39**	**0.03**

Logistic regression analysis with log-additive model in group “All”, and dominant model in groups “Dentists” and “Teachers”.

ORs and 95% CIs are adjusted for age, occupation and body mass index (BMI) in group “All” and for age and BMI in groups “Dentists” and “Teachers”.

NCBI-population is 1000 genomes.

HapMap population is CEU: Utah residents with Northern and Western European ancestry from the CEPH collection.

We found no statistically significant associations between the SNPs in the *GDF5*, *HFE* and *ESR1* genes and hand OA. Linkage disequilibrium was very strong between the two *COG5* SNPs (rs3757713 and rs3815148) as well as the two *ESR1* SNPs (*PvuII* rs2234693 and *XbaI* rs9340799) (D’ = 0.95, p<0.0001 and D’ = 0.99, p<0.0001, respectively). Haplotypes comprised of the two *COG5* SNPs or of the two *ESR1* SNPs were not associated with hand OA.

We found an interaction between the *ESR1* rs9340799 SNP and occupation (p = 0.0064) in relation to symptomatic DIP OA. The teachers with the minor allele were at an almost three-fold increased risk of symptomatic DIP OA (OR = 2.84, 95% CI 1.25–6.48) while the dentists carrying the allele were at a lower risk (OR = 0.50, 95% CI 0.19–1.33) than those with the major allele. Adjustment for the use of hormone therapy did not affect the observed associations.

### Exploratory Analyses of Nine OA-associated SNPs

Of nine SNPs associated with different OA phenotypes (knee, hip, spine and multiple joints) in previous studies, only the *TGFB1 Leu10Pro* (rs1800470) SNP was associated with symptomatic DIP OA (Table 3, OR = 1.84, 95% CI 1.16–2.91). We observed a suggestive (p = 0.047) interactive effect between the *PTGS2* rs4140564 SNP and occupation in relation to ROA. The teachers with the minor allele were at a 1.44-fold (95% CI 0.69–3.02) increased risk, and the dentists were at a lower risk (OR = 0.40, 95% CI 0.13–1.21) (Table 2).

### Gene-gene Combination Effects and Interaction

To evaluate possible gene-gene combination effects and interactions, we selected two SNPs from the genes located on chromosome 7 (*COG5* rs3757713 and *BCAP29* rs10953541) and two SNPs from the genes located on chromosome 6 (*HFE* rs179945 and *ESR1* rs9340799) that showed relatively stronger associations with our hand OA phenotypes. We found a statistically significant interaction (p = 0.034) between the *COG5* rs3757713 and *BCAP29* rs10953541 SNPs and a suggestive interaction (p = 0.096) between the *HFE* rs179945 and *ESR1* rs9340799 SNPs (Table 4). Carriage of the *COG5* rs3757713 C allele increased the risk of ROA only in women with the *BCAP29* rs10953541 CC genotype. However, the likelihood of ROA in the carriers of the minor allele of either SNPs (rs3757713 or rs10953541) was 1.49 (95% CI 1.07–2.06) times higher than that in the non-carriers of these alleles. The carriage of the *HFE* rs179945 G allele increased the risk of symptomatic DIP OA in women homozygous for the *ESR1* rs9340799 major allele (AA genotype). Carriage of the minor allele of either SNP (rs179945 or rs9340799) was associated with an approximately two-fold increased risk of symptomatic DIP OA (OR = 2.12, 95% CI 1.28–2.50).

**Table 4:** Individual and joint effect of selected SNPs on ROA and symptomatic DIP OA.

			ROA				Symptomatic DIP OA			
			N	OR	95% CI	p-value*	N	OR	95% CI	p-value*
*COG5-BCAP29*	*COG5* rs3757713 C-allele carriage	*BCAP29* rs10953541 T-allele carriage				0.034				0.54
	No	No	74/277	1.00			21/277	1.00		
	Yes	No	11/27	**2.57**	**1.08**–**6.10**		3/27	2.20	0.57–6.40	
	No	Yes	30/88	1.53	0.89–2.65		8/88	1.24	0.51–3.00	
	Yes	Yes	45/149	1.24	0.78–1.97		17/149	1.62	0.80 –3.26	
Carriage of COG5 C or BCAP29 T allele			86/264	**1.48**	**1.07**–**2.06**		28/264	1.55	0.93–2.58	
*HFE*–*ESR1*	*HFE* rs179945 G–allele carriage	*ESR1* rs9340799 C–allele carriage				0.19				0.096
	No	No	63/237	1.00			15/237	1.00		
	Yes	No	21/60	1.60	0.84–3.03		9/60	**3.01**	**1.19**–**7.58**	
	No	Yes	58/180	1.34	0.86–2.11		19/180	1.86	0.89–3.87	
	Yes	Yes	18/65	1.16	0.61–2.21		6/65	1.77	0.63 –4.95	
Carriage of HFE G or ESR1 C allele			97/305	1.35	0.98–1.86		34/305	**2.12**	**1.28**–**3.50**	

*p-value for interaction; ORs and 95% CIs are adjusted for age, occupation and body mass index.

## Discussion

In this study we attempted to replicate some previously reported genetic associations with hand OA. Of the two SNPs identified in GWAS, we found a statistically significant association between the *A2BP1* rs716508 SNP and radiographic hand OA. In addition, we observed evidence of an interactive effect between the *COG5* rs3757713 and the *BCAP29* rs10953541, and radiographic hand OA, and between the *HFE* rs179945 and the ESR1 rs9340799 and symptomatic DIP OA. Moreover, our exploratory analysis of the SNPs in nine selected genes that have a hypothetical association with hand OA provided evidence of an association between the *TGFB1 Leu10Pro* (rs1800470) SNP and symptomatic DIP OA.

In order to succeed in the replication of genotype-phenotype associations, both the initial and replication studies should preferably have the same gene variants, disease phenotypes, genetic models, and population segment (*e.g*. the same gender), and should be adjusted for the same confounding factors. We selected two hand OA phenotypes (ROA in at least three finger joints and symptomatic DIP OA), that are alike or very similar to those in the discovery studies. Both our phenotypes were defined on the basis of a K–L score of grade 2 or more (at least mild OA), which has been commonly used for classification in other studies [24], [26], [39]. Radiographic hand OA in our study was based solely on radiographic evidence of joint destruction, while symptomatic DIP OA was defined on the basis of the simultaneous presence of radiographic changes and pain in the most commonly affected joint (DIP).

GWAS with two discovery and four replication samples reported an association between the rs716508 SNP located in the first intron of the *A2BP1* gene and hand OA in the population of European ancestry [18]. Meta-analysis suggested that the minor allele of this SNP diminished the risk of hand OA by 33–41%, with the protective effect larger for the severe hand OA phenotype (at least three joints affected). In line with the initial study, we observed a 32% reduction in the risk of radiographic OA per minor allele. However, we found no association between the rs716508 SNP and symptomatic DIP OA.

The *A2BP1* gene is known to be associated with several human diseases, such as autism [40], cancer [41] and obesity [42], though its role in the development of hand OA needs further exploration. Since the *A2BP1* gene is abundantly expressed in the skeletal muscle [43], it is possible that the association between the rs716508 SNP and hand OA could be via muscle strength. We have earlier reported an association between pinch grip strength and symptomatic ROA, but radiographic findings or pain were not associated with pinch grip strength [44]. In the present study, the rs716508 SNP was not associated with pinch grip strength and the odds of ROA remained unchanged after adjustment for pinch grip strength (data not shown).

Although we failed, in the sample of Finnish female dentists and teachers, to replicate the associations of the other SNPs with reported effects on hand OA, the effect sizes of the risk alleles on ROA and symptomatic DIP OA were similar to those found in the initial studies. Reasons for the lack of replication could be due to differences in phenotypes, in minor allele frequencies, and the high likelihood of false negative findings, because our study was not large enough to detect very small effects.

Multiple susceptibility variants with small effect sizes may also interact with each other or with environmental factors in their influence on hand OA predisposition.

In agreement with this, our result suggests that the effect of the rs3757713 SNP (that is in complete linkage with rs3815148) on hand ROA might be diluted by the carriage of the *BCAP29* rs10953541 minor allele. The *COG5* and *BCAP29* genes together with the other four genes (*PRKAR2B*, *HBP1*, *GPR22* and *DUS4L*) are located on chromosome 7q22 within a block with high-linkage disequilibrium. The GWAS meta-analyses of knee OA showed that this LD block contains an OA susceptibility locus [27]. Both genes were expressed in OA joint tissue but were down-regulated in OA cartilage compared to normal control cartilage [45].

In addition, we found that the effect of the *HFE* rs1799945 on symptomatic DIP OA was diluted by the carriage of the *ESR1* rs9340799 minor allele. Both genes may play a role in hand OA, which is particularly prevalent among women, especially post menopause. The rs1799945 variant in the *HFE* (hemochromatosis Fe) gene is the most common genetic factor in hereditary hemochromatosis. A high risk of developing OA has been observed in iron overload patients, who carry an *HFE* mutation [46]. Increasing evidence shows that a link between defective iron metabolism and tissue responses may drive the OA phenotype [47]. A significant increase occurs in women’s iron levels during menopausal transition [48]. Therefore, iron increase could be a risk factor in age-related OA. Candidate gene association studies consistently showed associations between mutations within the *HFE* gene and different hand OA phenotypes [49]. Inverse changes have been observed between iron and estrogen levels in healthy women during menopausal transition [50].

The estrogen receptor 1 alpha (ESR1) gene has been investigated in several genetic studies of knee OA, but very little in relation to hand OA. Valdes *et al.* [51] reported an association between the gene and clinical knee OA in women but not in men. Bergink *et al.* [52] found an association between the *ESR1* haplotype and radiographic knee OA in elderly men and women. One study of Japanese women that investigated the association of *PvuII* (rs2234693) and *XbaI* (rs9340799) SNPs in the *ESR1* gene with generalized hand OA observed an approximately two-fold increase in the risk of severe generalized OA among participants carrying heterozygous genotypes for both rs2234693 and rs9340799 loci [53]. However, these associations have not been replicated in European descent for hand OA [26]; in European descent the *PvuII* variant allele has been associated with a reduced risk of hip OA in elderly women [54].

The SNPs selected for exploratory analysis in our study are located in potential candidate genes for OA identified by GWAS. We found that the variants in the *BCAP29, DIO2, DUS4L, DVWA, HLA, PARD3B, PTGS2, TGFB1* and *TRIB1* genes were associated with OA across multiple sites [27]–[33]. We also discovered that the *TGFB1* (rs1800470, formerly known as rs1982073) variant allele (*Leu10*) was associated with a substantially increased risk of symptomatic DIP OA, but not radiographic OA. Transforming growth factor-β (TGFB) is a multifunctional growth factor with widespread effects on multiple tissues, including an important role in cartilage matrix metabolism. Elevated TGFB1 levels have been identified in the synovial fluid of patients with OA [55], and levels after meniscectomy are reflective of future bone and cartilage changes [56]. The functional role of the *Leu10Pro* polymorphism remains largely unknown, but some evidence exists that it affects TGFB1 secretion and functions in hepatic cells [57]. Earlier, this polymorphism was believed to be associated with a radiographic spinal OA among Japanese women [32].

Our study had both strengths and limitations. The main limitation was the relatively small sample size, due to which the study had low power to detect small effects. One major strength was that it consisted of random samples of ethnically relatively homogenous Finnish origin. The Finnish population is one of the best-studied genetic isolates, which originated from a small founder population some 2000 years ago. Therefore, the Finnish population has a relatively homogenous gene pool [58] offering optimal material for association studies. We also chose to have only women in our study, thus findings that might be restricted to men were left undetected. Moreover, the age range (45–63 years) of the participants was selected to cover the age of occupationally active women, whose prevalence of hand OA is rapidly increasing. Finally, dentists and teachers have a similar level of education, although occupational exposures related to hand use, and lifestyle factors such as obesity, differed.

The current findings were also unlikely to have been influenced by selection bias. The prevalence of the studied hand OA phenotypes was similar to those observed in other studies [59], [60]. Yet, the ‘healthy worker effect’ may have led to an underestimation of associations between SNPs and hand OA, especially for symptomatic DIP OA. Dentists suffering from severe pain in the hand joints may not be able to continue in their profession and therefore were outside the occupationally active group that was our target population. In contrast, teachers with symptomatic hand OA may remain active in their profession. Indeed, occupation-stratified analyses revealed that most of the studied SNPs had a different effect on hand OA in the two occupational groups.

To summarize, by replicating and partly confirming earlier studies of OA susceptibility polymorphisms, our results further support the theory that the *A2BP1* and *TBGF1* genes are hand OA susceptibility genes. OA is a globally significant medical, social, and economic problem and therefore merits attention in order to develop better prevention methods and therapies that can be applied worldwide. The identification of susceptibility genes is a promising basis for OA prevention and treatment.

## Supporting Information

Table S1 **Description of SNPs selected for replication and exploratory analyses.** References for Table S1 [61], [62].
(DOC)
