# Supplementary material for: Genetic Influences on Hand Osteoarthritis in Finnish Women – A Replication Study of Candidate Genes
Source: PLoS One. 2014 May 13;9(5):e97417. doi: 10.1371/journal.pone.0097417 (PMC4019597; doi:10.1371/journal.pone.0097417)
Supplement: Table S1 — Description of SNPs selected for replication and exploratory analyses. References for Table S1 [61], [62]. (DOC) [file pone.0097417.s001.doc]

| **Gene** | **refSNP** | **Chr** | **Function** | **F-SNP #** | **Study type** | **OA site** | **Sex** | **Reference** | **OA pathway*** | **Study population** | **Effect size OR (95% CI) (OA site, gender)** |
| --- | --- | --- | --- | --- | --- | --- | --- | --- | --- | --- | --- |
| **SNPs selected for replication analysis** | | | | | | | | | | | |
| A2BP1 | rs716508 | 16 | intron | 1 | GWAS | hand | women | Zhai | BMD, HGS | TwinsUK, Rotterdam, Chingford, Chuvasha | p=1.8x10^-5^ (hand, female, meta-analysis) |
| COG5 | rs3757713 | 7 | intron | NA |  |  |  | Kerkhof |  |  | LD with next one |
|  | rs3815148 | 7 | intron | NA | GWAS | hand and/or knee | both | Kerkhof | cartilage, GPR22 expression, osteophytes | Rotterdam, deCODE, TwinsUK, Framingham, Chingford, Oxford, Nottingham, Greek, Spanish, GARP, SOF, MrOS | 1.14 (1.09-1.19) p=8.0x10^-8^ (hand and/or knee, both, meta-analysis) |
| GDF5 | rs143383 | 20 | UTR-5 | 5 | Case-control | knee, hip | both | Miyamoto | cartilage | Japanese, Chinese | 1.79 (1.53-2.09) (hip, both), 1.54 (1.22-1.95) (knee, both) |
|  |  |  |  |  | Cohort | knee, hand | women | Vaes |  | Rotterdam | 37% lower risk (p = 8x10^-6^ ) (hand, female) |
|  |  |  |  |  | Meta-analysis | knee, hip, hand | women | Evangelou |  | TwinsUK, Rotterdam, deCODE, GARP, Gonzalez, Kujala | 1.11 (0.94-1.31), p=0.22 (hand, female) |
| HFE | rs1799945 | 6 | missense | 2,3,4,5 | Case-control | knee, hand | both | Carroll | Age-related onset of OA | Australia | p=0.0001 (MCP 2,3 joints, both) |
| ESR1 | rs2234693 | 6 | intron | 1 | Cohort | knee, hip | women | Riancho | Age-related onset of OA, hormonal status | Spanish, Oxford | 0.76 (0.59-0.97) (knee, female) |
|  | rs9340799 | 6 | intron | 1 | Cohort | finger joints | women | Wise |  | Framingham | 0.92 (0.69-1.22) (hand, female) |
| **SNPs selected for exploratory analysis** | | | | | | | | | | | |
| BCAP29 | rs10953541 | 7 | intron | 1 | GWAS | knee | both | Evangelou | cartilage | deCODE, Rotterdam, Framingham, TwinsUK, arcOGEN, Greek, Spanish, Finnish, Nottingham, Chingford, GARP, Estonian, Swedish | 1.17 (1.10-1.23) (knee, both) |
| DUS4L | rs4730250 | 7 | intron | NA | GWAS | knee | both | Evangelou | cartilage |  | 1.17 (1.11-1.24) (knee, both) |
|  |  | 7 |  |  | Linkage | DIP | women | Hunter |  | Framingham |  |
| DIO2 | rs225014 | 14 | missense | 2,3,4,5 | Linkage/GWAS | Symptomatic OA at multiple joint sites (siblings) | both | Meulenbelt | osteophytes, thyroid hormone | GARP, UK, Rotterdam, Japanese | 1.79 (1.37-2.34) (hip, female) |
| DVWA | rs7639618 | 3 | ncRNA~ | 1,2,3,4,5 | GWAS | knee | both | Miyamoto | cartilage | Japanese, Chinese | 1.54 (1.32-1.81) (knee, both) |
|  |  | 3 |  |  | Linkage | hand | both | [Greig](http://www.ncbi.nlm.nih.gov/pubmed?term=Greig C%5BAuthor%5D&cauthor=true&cauthor_uid=16504993) |  | Nottinghamshire UK |  |
| HLA | rs10947262 | 6 | intron | 1,3 | GWAS | knee | both | Nakajima | immunologic | Japanese, Spanish, Greek | 1.31 (1.20-1.44) (knee, both) |
| PARD3B | rs1207421 | 2 | intron | 1 | GWAS | knee | women | Valdes | inflammatory | Nottingham, Chingford, TwinsUK, US, Rotterdam | 1.40 (1.20-1.62) (knee, both, meta-analysis) |
|  |  | 2 |  |  | Linkage | hand | women | Livshits |  | TwinsUK |  |
| PTGS2 | rs4140564 | 1 | intergenic | NA | GWAS | knee | women | Valdes | inflammatory | Nottingham, Chingford, TwinsUK, US, Rotterdam | 1.55 (1.30-1.85) (knee, both, meta-analysis) |
|  |  | 1 |  |  | Linkage | DIP | women | [Hunter](http://www.ncbi.nlm.nih.gov/pubmed?term=Hunter DJ%5BAuthor%5D&cauthor=true&cauthor_uid=15334462) |  | Framingham |  |
| TGFB1 | rs1800470 /rs1982073 | 19 | missense | 1,2,3,4,5 | Case-control | spine | women | Yamada | BMD | Japanese | 2.3, p=0.04 (spine, female) |
|  |  | 19 |  |  | Linkage | knee, hand | women | Livshits |  | TwinsUK |  |
| TRIB1 | rs4512391 | 8 | intergenic | 5 | GWAS | knee, hip | both | Panoutsopoulou | lipid metabolism | UK, replication in deCODE, Framingham, Rotterdam and TwinsUK | 1.23 (1.13-1.33) (knee, both) 1.17 (1.10-1.25) (knee and hip, both) |
|  |  | 8 |  |  | Linkage | DIP | both | [Greig](http://www.ncbi.nlm.nih.gov/pubmed?term=Greig C%5BAuthor%5D&cauthor=true&cauthor_uid=16504993) |  | Nottinghamshire UK |  |

**Table S1.** Description of SNPs selected for replication and exploratory analyses.

# coding: 1 transcriptional regulation; 2 protein coding, deleterious; 3 splicing regulation, changed; 4 post translation; 5 conserved

**~** non-coding RNA

*BMD ‒ bone mineral density; HGS ‒ hand-grip strength;
